# Characterizing and assessing vision‐related quality of life among patients discontinued treatment for neovascular age‐related macular degeneration

**DOI:** 10.1111/aos.70044

**Published:** 2025-12-15

**Authors:** Noreddin Shekho, Anna Stage Vergmann, Frederik Nørregaard Pedersen, Lonny Stokholm, Benjamin Sommer Thinggaard

**Affiliations:** ^1^ Department of Ophthalmology Odense University Hospital Odense Denmark; ^2^ Department of Clinical Research University of Southern Denmark Odense Denmark

**Keywords:** anti‐VEGF, neovascular age‐related macular degeneration (nAMD), patient‐reported outcomes, treatment discontinuation, vision‐related quality of life (VRQoL)

## Abstract

**Purpose:**

This study characterized and assessed vision‐related quality of life (VRQoL) in patients with neovascular age‐related macular degeneration (nAMD) who discontinued treatment with intravitreal anti‐vascular endothelial growth factor (anti‐VEGF), comparing them to those undergoing treatment. Secondarily, it explored reasons for treatment discontinuation against medical advice.

**Methods:**

This survey‐based cross‐sectional study used data collected for the Danish study, “Identification of Patient‐Reported Barriers in Treatment for nAMD” (I‐OPTA) at Odense University Hospital, Denmark. I‐OPTA included a self‐developed questionnaire and the National Eye Institute Visual Function Questionnaire‐25 (NEI‐VFQ‐25). Main outcomes were demographics, treatment details, NEI‐VFQ‐25 scores, and reasons for treatment discontinuation against medical advice. Linear regression models investigated the impact of variables on the composite NEI‐VFQ‐25 score.

**Results:**

The study included 172 (32.6%) patients who had discontinued treatment and 356 (67.4%) patients who were undergoing treatment; 10 (5.8%) discontinued against medical advice. Discontinued patients were older (median 81.0 years, *p*‐value = 0.004), had lower best‐corrected visual acuity (BCVA) in the worse‐seeing eye (*p*‐value<0.001), had a shorter treatment duration (*p*‐value = 0.001) and lived alone (*p*‐value = 0.044). Discontinued patients showed lower scores in all NEI‐VFQ‐25 domains except ocular pain. Higher BCVA correlated with a higher composite score of NEI‐VFQ‐25. Reasons for discontinuation against medical advice included treatment burden and perceived inefficacy.

**Conclusion:**

Patients who discontinued treatment for nAMD report lower VRQoL, with lower BCVA in the worse‐seeing eye, older age, living alone, and unilateral treatment possibly contributing to treatment discontinuation. Future studies on visual acuity and retinal fluid in this group could guide decisions on treatment discontinuation, emphasizing patients' quality of life.

## 1 INTRODUCTION

Neovascular age‐related macular degeneration (nAMD) is one of the leading causes of visual impairment among older adults in developed countries (Wong et al., 2014). nAMD impacts central vision by the abnormal growth of vessels into the subretinal space beneath the macula, causing bleeding and intraretinal oedema (Rozing et al., 2020). Ageing is the main risk factor for developing nAMD and the 15‐year incidence of developing nAMD is 6.8% in persons aged 49 years or older (Joachim et al., 2015; Rozing et al., 2020). This makes nAMD a common disease, and its prevalence is expected to increase due to a high proportion of elderly in developed countries (Chopdar et al., 2003).

Loss of central vision among patients with nAMD affects many aspects of life, especially their independence in performing everyday tasks (Vu et al., 2023). Since the introduction of injections with intravitreal anti‐vascular endothelial growth factor (anti‐VEGF), the incidence rate of blindness due to nAMD has been reduced by approximately 50% (Finger et al., 2020). Furthermore, 12 months of treatment with anti‐VEGF has demonstrated an improvement in vision‐related quality of life (VRQoL) among patients with nAMD (Finger et al., 2020). Various treatment regimens with anti‐VEGF are employed and treatment is not always lifelong (Yeung et al., 2021). The Department of Ophthalmology at Odense University Hospital, Denmark, utilized 'Observe and Plan' treatment regimen for nAMD, initiating therapy with three injections at four‐week intervals, followed by an observational period with regular monitoring. Individualized intervals between injections are determined based on the outcome observed during this period. Similar to other countries, there are no established guidelines for discontinuing treatment with anti‐VEGF injections for nAMD (Yeung et al., 2021). This reflects the national and international lack of consensus on when to discontinue anti‐VEGF therapy for nAMD, as the decision often involves a shared assessment between the clinician and the patient (Yeung et al., 2021).

Numerous studies have examined visual function and assessed VRQoL among patients with nAMD who were undergoing treatment with anti‐VEGF (Fenwick et al., 2018; Marakis et al., 2022). However, no prior research has comprehensively characterized and assessed VRQoL among patients with nAMD who had discontinued treatment with anti‐VEGF. This gap in knowledge limits our understanding of the full impact of nAMD on patients' VRQoL.

This study is based on questionnaire responses from a Danish study entitled *“Identification of Patient‐Reported Barriers in Treatment for nAMD”* (I‐OPTA) (Thinggaard, Hansen, et al., 2024). The I‐OPTA study comprises several components, including the National Eye Institute Visual Function Questionnaire‐25 (NEI‐VFQ‐25) (Mangione et al., 2001), as well as self‐developed items concerning patient characteristics, use of preventive measures, and knowledge of treatment‐related complications (Thinggaard et al., 2025; Thinggaard, Hansen, et al., 2024; Thinggaard, Subhi, et al., 2024).

The primary purpose of this study was to characterize and assess VRQoL in patients with nAMD who had discontinued treatment with anti‐VEGF, and compare them to patients with nAMD who were undergoing treatment with anti‐VEGF. Secondarily, we explored the reasons for treatment discontinuation against medical advice to identify potential areas for enhancing patient experience with anti‐VEGF therapy.

## 2 METHODS

This was a cross‐sectional survey‐based study utilizing data from I‐OPTA, which collected data from patients with nAMD (Thinggaard, Hansen, et al., 2024). I‐OPTA utilized a combination of questionnaires to assess visual function, quality of life, and patient characteristics (Thinggaard, Hansen, et al., 2024). Its design and methodology, including a detailed description of the different recruitment strategies, have been described in extensive detail (Thinggaard, Hansen, et al., 2024).

### 2.1 Participants and data collection

The population of this study consisted of two patient groups recruited at the Department of Ophthalmology at Odense University Hospital, Denmark: (1) patients with nAMD who were undergoing treatment with anti‐VEGF, recruited in September 2023 and October 2023. (2) Patients with nAMD who had discontinued treatment with anti‐VEGF were defined as those who either reported they had discontinued treatment or those who were unsure but had not received an intravitreal anti‐VEGF injection for at least 4 months. We asked patients who had discontinued treatment if they did so against medical advice. Patients who answered “yes” were asked to provide a reason for their decision. For those who did not discontinue treatment against medical advice, we asked if they had discontinued treatment following a doctor's recommendation. Patients who answered “don't know” or “no” to that question were then asked to explain their reason for discontinuing treatment. Patients who had discontinued treatment were recruited from December 2022 to March 2023.

Patients who agreed to participate received a link to the survey and comprehensive information via Digital Post, which is a part of the cross‐governmental digital service infrastructure in Denmark that allows public authorities to communicate digitally with citizens and businesses through enhanced security layers (Agency for Digital Governmen, 2025). Patients, who were exempted from using Digital Post, such as those with severe visual impairments, had the opportunity to answer the questionnaire through a telephone interview. Patients with severe Alzheimer's disease and individuals who were deaf‐blind were excluded from participation in the study.

### 2.2 I‐OPTA questionnaire

I‐OPTA questionnaire consisted of five different questionnaires (Thinggaard, Hansen, et al., 2024). This study focused on data collected using the self‐developed background questions and NEI‐VFQ‐25 (Mangione et al., 2001; Thinggaard, Hansen, et al., 2024). The self‐developed background questions collected various demographic and informational data about the patient, including age, biological sex, treatment for nAMD, home‐to‐treatment travel time, marital status, retirement and living situation. The self‐developed background questions were developed by experts in nAMD and questionnaire methodology, informed by insights from a prior interview‐based study (Thinggaard et al., 2023; Thinggaard, Hansen, et al., 2024). The validation of the self‐developed questions involved six steps: defining constructs through a literature review, expert reviews, focus group discussions with the patient and public involvement board, pilot testing with 12 non‐nAMD and seven nAMD patients, cognitive interviews using a retrospective probing technique for feedback, and survey revisions (Thinggaard, Hansen, et al., 2024). Meanwhile, the NEI‐VFQ‐25 is a commonly used, reliable and valid questionnaire for assessing patient‐reported visual function and VRQoL among patients with AMD (Mangione et al., 2001; Sørensen et al., 2011).

### 2.3 Variables

The NEI‐VFQ‐25 questionnaire comprises 25 questions divided into 12 domains (general health, general vision, ocular pain, near activities, distance activities, social functioning, mental health, role difficulties, dependency, driving, colour vision, and peripheral vision). All domains are included in the composite score except for the general health domain, which is not a vision‐related domain (Mangione et al., 2001).

The number of questions differs according to domain, and the score for each domain is calculated from the mean scores of its questions. Unanswered questions are excluded from the mean calculation of the domain score. The scores range from 0 to 100, where higher values represent better visual function related to VRQoL.

The variable ‘treatment laterality’ refers to whether the patient was treated bilaterally (in both eyes) or unilaterally (in one eye) with anti‐VEGF injections. This variable reflects if the patient had nAMD in one or both eyes. The variable ‘living situation’ inquired about whether the patient lived alone or not. The variable ‘retired’ inquired about whether the patient was retired or not. The variable ‘telephone interview’ indicated whether the patient completed the questionnaire via a telephone interview. Age was calculated based on the patient's date of birth and the survey response date. ‘Home‐to‐treatment travel time’ had four answer categories (<30 min, 30–60 min, 60–90 min and >90 min). To improve accessibility analysis and ensure sufficient statistical power, these categories were re‐categorized (<30 min and ≥ 30 min). This approach is supported by previous research, which has demonstrated that travel time within 30 minutes is a significant indicator of healthcare accessibility (Bosanac et al., 1976). ‘Marital status’ initially had five response categories: Married, Widowed, Divorced, Never Married, and Other. To ensure sufficient statistical power and create groups of approximately equal size, these categories were re‐categorized into three: Married, widowed/widower, and divorced/never Married/other.

The variable ‘duration since the last injection’ was calculated for patients who had discontinued treatment. It represents the time elapsed in years between the date of their last anti‐VEGF treatment session and the date they completed the questionnaire. The variable ‘treatment duration’ was calculated differently for patients who were undergoing treatment and those who had discontinued treatment. For patients who were undergoing treatment, it was calculated from the year they reported starting treatment to the date they completed the questionnaire. For patients who had discontinued treatment, it was calculated based on the dates of their first and last treatment sessions.

Best‐corrected visual acuity (BCVA) was determined through a review of patient records and was assessed using a standard Snellen chart and documented in decimal notation. For patients who were undergoing treatment, a maximum interval of 8 weeks was allowed between the BCVA measurement and the response date. For those who had discontinued treatment, the final recorded BCVA at the time of discontinuation was used. For statistical analysis, the BCVA for each patient was categorized into two variables: BCVA in the best‐seeing eye and BCVA in the worse‐seeing eye.

### 2.4 Analysis

Baseline characteristics are presented as median or counts. Percentages were calculated separately within each patient group. Chi‐square tests of independence (χ^2^) were used to assess associations between categorical variables, and Mann–Whitney U tests were used to assess differences in the distributions of continuous variables. The unpaired t‐test was used to compare differences in scores of NEI‐VFQ‐25.

Linear regression models with bootstrapping were conducted to estimate regression coefficients with 95% confidence intervals between different variables and the composite score of the NEI‐VFQ‐25, separately for patients who were undergoing treatment and those who had discontinued treatment. Linear regression with a bootstrapping model was adjusted for biological sex, treatment laterality, age and BCVA in the best‐seeing eye. The selection of confounders was based on prior knowledge. The statistical significance was indicated by a *p*‐value less than 0.05 for the chi‐square test, the Mann–Whitney U test and the unpaired t‐test. For linear regression models, significance was indicated by a 95% confidence interval that did not include 0 (no difference in means).

### 2.5 Ethics

This study adheres to the ethical principles outlined in the Declaration of Helsinki. Prior to participating in the study, all participants provided informed consent. The necessary approvals to conduct this study were obtained from the data processing activities records of the Region of Southern Denmark (Journal no 22/10138).

## 3 RESULTS

Between September 29, 2023, and February 19, 2024, a total of 535 patients with nAMD responded to the I‐OPTA questionnaire. BCVA was successfully obtained for 506 of the 535 patients (94.6%). The median time gap between the BCVA record for patients who had discontinued treatment and the questionnaire mailing date was 2 years (Interquartile Range (IQR): 1–3 years). Seven patients were excluded due to incomplete responses to the NEI‐VFQ‐25 questionnaire. Of the 528 patients with nAMD, 356 (67.4%) were undergoing treatment with intravitreal anti‐VEGF injections, while 172 (32.6%) had discontinued treatment with intravitreal anti‐VEGF injections. Of patients who had discontinued treatment, 10 (5.8%) discontinued treatment against medical advice (Table 1).

**TABLE 1:** Descriptive characteristics of patients with nAMD who had discontinued treatment with anti‐VEGF (172) and patients who were undergoing treatment (356).

	Discontinued treatment	Undergoing treatment	*p*‐value
Number of patients, *n* (%)	172 (32.6)	356 (67.4)	‐
Age, median in years (IQR)	81.0 (76.5–86.7)	79.3 (75.0–84.1)	0.004
Biological sex, *n* (%)			0.305
Female	109 (63.4)	209 (58.7)	
Male	63 (36.6)	147 (41.3)	
BCVA, median in Snellen decimal (IQR)			
Best‐seeing eye	0.8 (0.32–1)	0.8 (0.63–1)	0.135
Worse‐seeing eye	0.1 (0–0.4)	0.4 (0.13–0.63)	<0.001
Marital status, *n* (%)			0.062
Married	80 (46.5)	200 (56.2)	
Widow/widower	62 (36.1)	115 (32.3)	
Divorced/never married/other	30 (17.4)	41 (11.5)	
Living situation, *n* (%)			0.044
Not living alone	87 (50.9)	213 (59.8)	
Living alone	85 (49.4)	143 (40.2)	
Retired, *n* (%)	164 (95.4)	346 (97.2)	0.274
Treatment laterality, *n* (%)			0.041
Unilateral	116 (68.2)	210 (59.0)	
Bilateral	54 (31.8)	146 (41.0)	
Home‐to‐treatment travel time, *n* (%)			0.117
< 30 min	73 (42.4)	126 (35.4)	
≥ 30 min	99 (57.6)	230 (64.6)	
Telephone interview, *n* (%)	38 (22.1)	14 (3.9)	<0.001
Treatment duration, median in years (IQR)	3.3 (1–3)	4.6 (2–7)	0.001
Duration since the last injection, median in years (IQR)	2.8 (1–4)	‐	‐
Discontinued treatment against medical advice, *n* (%)	10 (5.8)	‐	‐

Abbreviations: Anti‐VEGF, anti‐vascular endothelial growth factor; BCVA, best‐corrected visual acuity (Snellen); IQR, interquartile range; nAMD, neovascular age‐related macular degeneration.

Patients with nAMD who had discontinued treatment were older (median age 81.0 years, IQR: 76.5–86.7 years) compared to those who were undergoing treatment (median age 79.3 years, IQR: 75.0–84.1 years; *p*‐value = 0.004, Mann–Whitney U test). The proportion of females did not significantly differ between the two patient groups (*p*‐value = 0.305, chi‐square test). Both the discontinued group (63.4% female) and those who were undergoing treatment (58.7% female) showed a higher representation of females. Patients who had discontinued treatment had significantly lower BCVA in the worse‐seeing eye (median 0.1 Snellen decimal, IQR: 0–0.4) compared to patients who were undergoing treatment (median 0.4 Snellen decimal, IQR: 0.13–0.63, *p*‐value <0.001, Mann–Whitney U test). A higher proportion of patients who had discontinued treatment lived alone (49.4% vs. 40.2%, *p*‐value = 0.044, chi‐square test) and utilized telephone interviews to complete the questionnaire (22.1% vs. 3.9%, *p*‐value <0.001). A higher proportion of patients who had discontinued treatment received anti‐VEGF injections unilaterally (68.2% vs. 59.0%, *p*‐value = 0.041). The median treatment duration with anti‐VEGF was shorter for patients who had discontinued treatment compared to those who were undergoing treatment (3.3 years, IQR: 1.0–3.0 years vs. 4.6 years, IQR: 2.0–7.0 years, *p*‐value = 0.001, Mann–Whitney U test). The median duration since the last injection for patients who had discontinued treatment was 2.8 years (IQR: 1.0–4.0 years). Chi‐square tests revealed no significant associations between the patient groups and marital status, home‐to‐treatment travel time or retirement status (Table 1).

Among the 10 patients who had discontinued treatment against medical advice (median age 82.5 years, IQR: 75.5–91.6 years), eight were females (80%) and seven received unilateral treatment (70%). The median treatment duration was 2.5 years (IQR: 1.0–3.0 years), and the median duration since the last injection was 2.5 years (IQR: 2.0–5.0 years). The median BCVA in the best‐seeing eye was 0.8 Snellen decimal (IQR: 0.5–0.8), and was 0.5 Snellen decimal (IQR: 0.02–0.63) in the worse‐seeing eye. Patients reported one to two reasons for discontinuing treatment against medical advice (Table 2). Some patients reported experiencing anti‐VEGF injections as painful or unbearable to go through, while others reported that the treatment was time‐consuming. Other patients doubted the efficacy of the treatment and some were satisfied with their current vision. COVID‐19 was also cited as a reason for discontinuing treatment against medical advice (Table 2).

**TABLE 2:** Reasons for treatment discontinuation against medical advice.

Question	Answer
What is the reason why you decided to stop treatment?	
	The patient was repeatedly assured that everything was fine, and therefore felt no need for further treatment.*
	Corona.
	Couldn't bear the thought of going through treatment.
	Didn't want injections. Was told there were limited prospects.
	I saw really well.
	It was too painful.
	No change in AMD.
	The treatment was traumatizing; it was an assault.
	I feel that treating age‐related ailments takes up too much of my life, and I would prefer to let nature take its course.
	Heard there was someone in the waiting room who had gotten 44 injections. Does it help? I chose to stop because there were risks with the treatments.

Abbreviations: AMD, age‐related macular degeneration; Corona, coronavirus disease (COVID‐19).

* An assistant wrote the quote during a phone interview.

### 3.1 NEI‐VFQ‐25 scores

**FIGURE 1:**
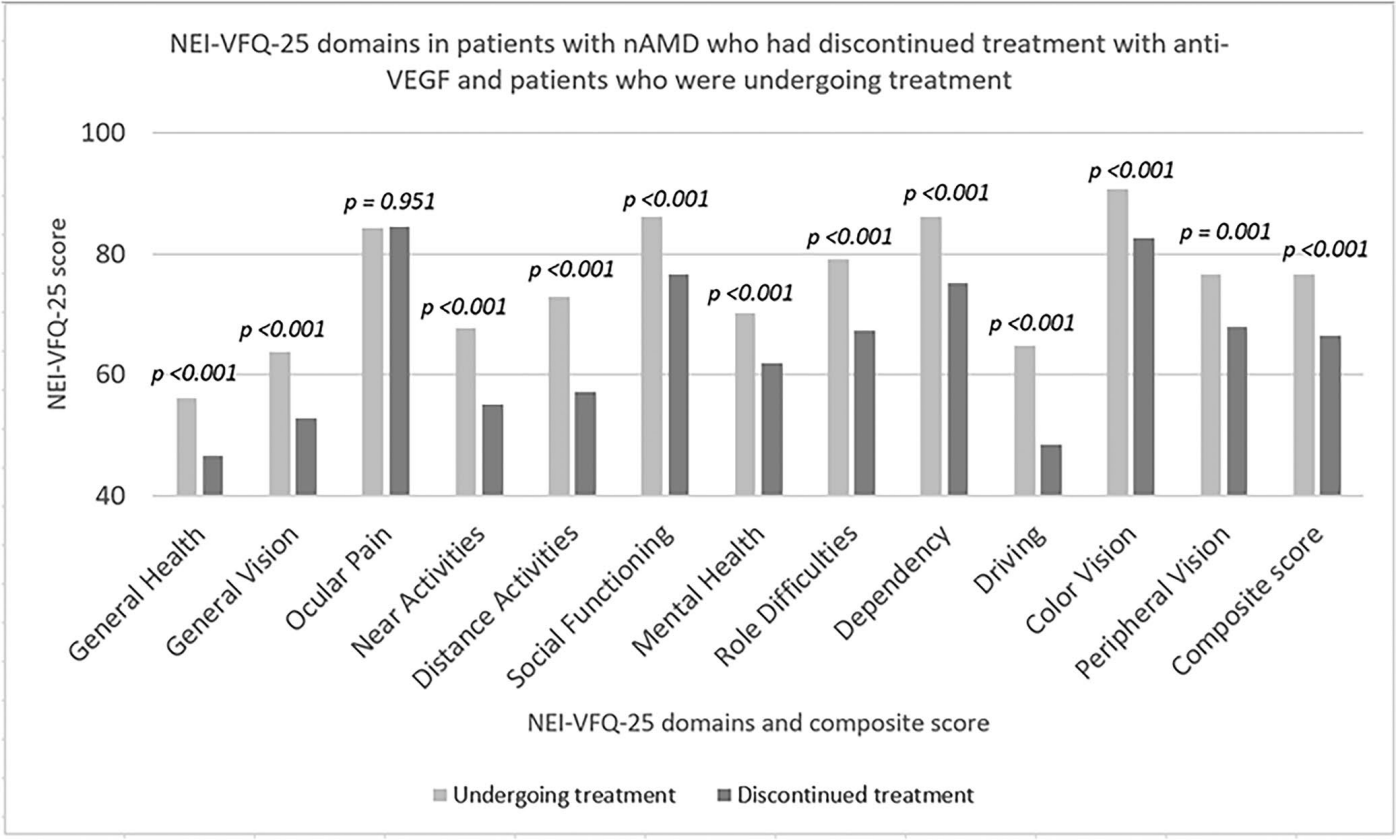
Mean scores of the NEI‐VFQ‐25 domains and composite scores in patients with nAMD who had discontinued treatment with anti‐VEGF (*n* = 172) and patients who were undergoing treatment (*n* = 356). Anti‐VEGF, anti‐vascular endothelial growth factor; nAMD, neovascular age‐related macular degeneration; NEI‐VFQ‐25, National Eye Institute Visual Function Questionnaire‐25.

Patients who had discontinued treatment exhibited significantly lower scores in all domains of the NEI‐VFQ‐25 except ocular pain compared to patients who were undergoing treatment (Figure 1). The three domains with the lowest scores in patients who had discontinued treatment were general health (mean: 46.66 vs. 56.04), general vision (mean: 52.91 vs. 63.88), and driving (mean: 48.38 vs. 64.89). On the other hand, the three domains with the highest scores were ocular pain (mean: 84.38 vs. 84.27), colour vision (mean: 82.70 vs. 90.59) and social function (mean: 76.53 vs. 86.10). A similar pattern was found among patients who were undergoing treatment (Figure 1).

**FIGURE 2:**
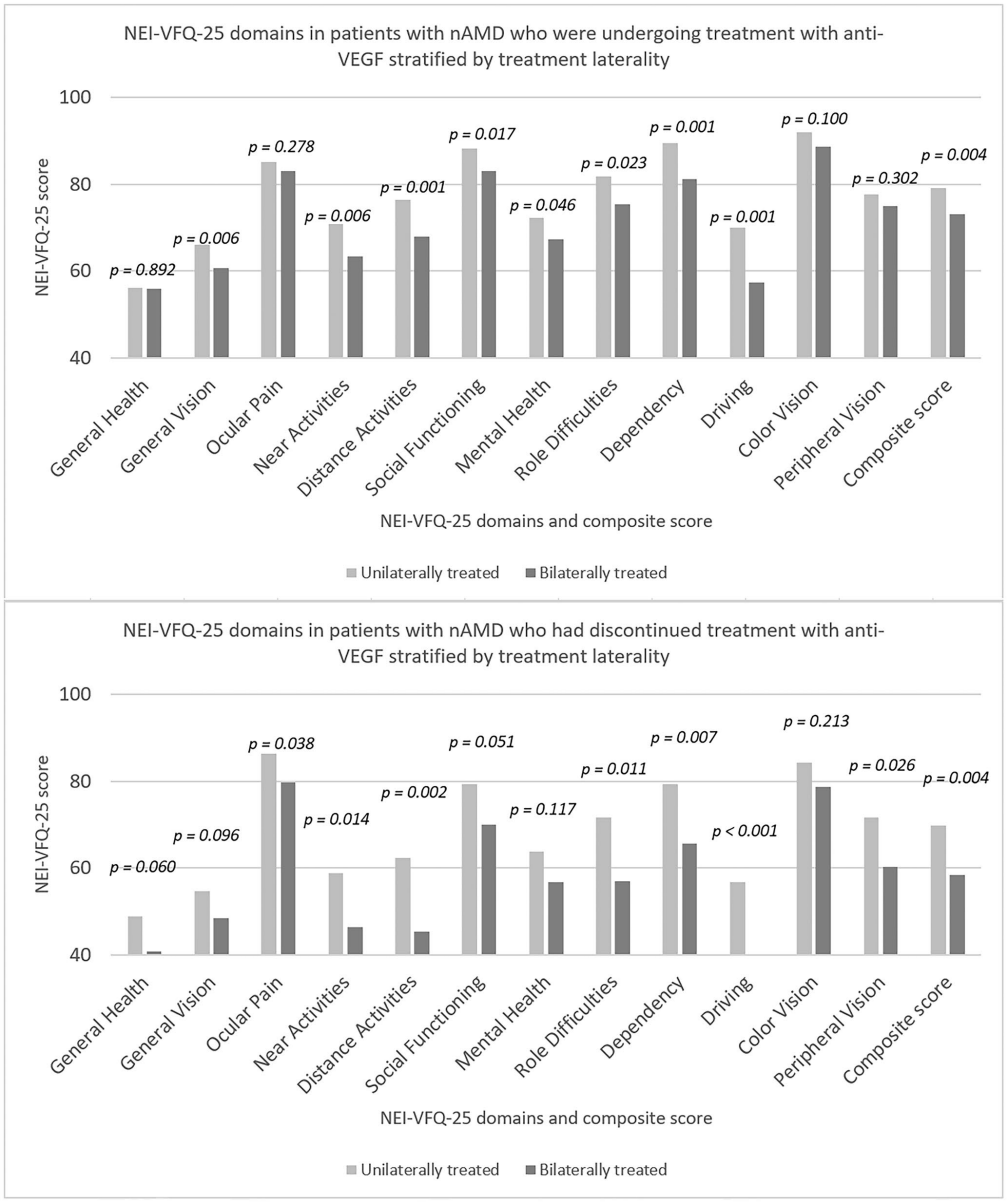
Mean scores of NEI‐VFQ‐25 domains and composite scores in patients with nAMD stratified by treatment laterality and treatment status. The groups include patients who were undergoing treatment (*n* = 356; 210 unilateral, 146 bilateral) and patients who had discontinued anti‐VEGF treatment (*n* = 172; 116 unilateral, 54 bilateral). Anti‐VEGF, anti‐vascular endothelial growth factor; nAMD, neovascular age‐related macular degeneration; NEI‐VFQ‐25, National Eye Institute Visual Function Questionnaire‐25.

When comparing the NEI‐VFQ‐25 domain scores, we found several differences between unilaterally and bilaterally treated patients. Among patients who had discontinued treatment, those treated unilaterally had significantly higher scores in ocular pain, near activities, distance activities, mental health, role difficulties, dependency, driving, and peripheral vision. Similarly, among patients who were undergoing treatment, those treated unilaterally demonstrated significantly higher scores in general vision, near activities, distance activities, social functioning, mental health, role difficulties, dependency, and driving (Figure 2).

In the adjusted linear regression model, age was significantly associated with lower composite scores of the NEI‐VFQ‐25 among patients who had discontinued treatment (β = −0.62, 95% Confidence Interval (CI): −1.07 to −0.17, linear regression model). This significant association was not found among patients who were undergoing treatment (β = −0.03, 95% CI: −0.29 to 0.23) (Table 3). BCVA was positively associated with NEI‐VFQ‐25 composite scores. Among patients who had discontinued treatment, a 0.1‐unit higher BCVA (Snellen decimal) corresponded to 3.73 points higher composite scores when measured in the best‐seeing eye (β = 37.28, 95% CI: 26.79–47.78) and 2.70 points in the worse‐seeing eye (β = 27.02, 95% CI: 17.79–36.26). Among patients who were undergoing treatment, the corresponding differences were 3.48 points (β = 34.82, 95% CI: 27.57–42.08) and 2.46 points (β = 24.64, 95% CI: 18.57–30.71). Male patients exhibited significantly higher composite scores of the NEI‐VFQ‐25 on average compared to female patients among those who had discontinued treatment (β = 6.67, 95% CI: 0.49 to 12.84), and among those who were undergoing treatment (β = 3.62, 95% CI: 0.05 to 7.18). No significant associations were found for ‘marital status’, ‘living situation’, ‘treatment laterality’, ‘home‐to‐treatment travel time’, ‘treatment duration’ and ‘duration since the last injection’ in the adjusted linear regression model. However, the crude regression model indicated that bilaterally treated patients who had discontinued treatment had significantly lower NEI‐VFQ‐25 composite scores on average (β = −11.39, 95% CI: −19.35 to −3.44) and a negative association between being widowed or living alone and the NEI‐VFQ‐25 composite score was found in both patient groups (Supplementary Table S1).

**TABLE 3:** Adjusted β‐coefficients and 95% confidence intervals for the NEI‐VFQ‐25 composite score and various covariates, separately for patients with nAMD who had discontinued treatment with anti‐VEGF (*n* = 172; 116 unilateral, 54 bilateral) and patients who were undergoing treatment (*n* = 365; 210 unilateral, 146 bilateral).

Variable	Adjusted β‐coefficient [95% CI]	
	Discontinued treatment	Undergoing treatment
Age	−0.62 [−1.07, −0.17]	−0.03 [−0.29, 0.23]
Biological sex		
Female	Ref	Ref
Male	6.67 [0.49, 12.84]	3.62 [0.05, 7.18]
BCVA		
Best‐seeing eye	37.28 [26.79, 47.78]	34.82 [27.57, 42.08]
Worse‐seeing eye	27.02 [17.79, 36.26]	24.64 [18.57, 30.71]
Marital status		
Married	Ref	Ref
Widow/widower	−2.75 [−10.89, 5.39]	−0.82 [−5.07, 3.44]
Divorced/never married/other	4.93 [−3.80, 13.65]	2.16 [−2.57, 6.88]
Living situation		
Not living alone	Ref	Ref
Living alone	2.56 [−4.30, 9.41]	0.71 [−2.97, 4.38]
Treatment laterality		
Unilateral	Ref	Ref
Bilateral	−2.40 [−9.54, 4.74]	−2.41 [−6.34, 1.53]
Home‐to‐treatment travel time		
< 30 min	Ref	Ref
≥ 30 min	−4.49 [−10.41, 1.43]	−1.17 [−4.72, 2.38]
Treatment duration	‐	−0.38 [−0.91, 0.15]
Duration since the last injection	0.27 [−1.78, 1.84]	‐

Abbreviations: Anti‐VEGF = anti‐vascular endothelial growth factor; BCVA, best‐corrected visual acuity (Snellen); CI, confidence interval; nAMD, neovascular age‐related macular degeneration; NEI‐VFQ‐25, National Eye Institute Visual Function Questionnaire‐25; Ref, reference.

## 4 DISCUSSION

Although patients with nAMD who had discontinued treatment with anti‐VEGF had a shorter treatment duration, they exhibited significantly lower VRQoL compared to patients who were undergoing treatment. This study revealed a high rate of treatment adherence among patients who had discontinued treatment with anti‐VEGF, with only a small percentage discontinuing treatment against medical advice. Factors such as lower BCVA in the worse‐seeing eye, older age, living alone and unilateral treatment were correlated with treatment discontinuation. Reasons for treatment discontinuation against medical advice included concerns about treatment burden and perceived efficacy.

NEI‐VFQ‐25 scores for patients who were undergoing treatment in our study were similar to those in other studies (Jelin et al., 2019; Patil et al., 2022; Zhu et al., 2017). However, the lack of data on the status and type of retinal fluid, which are factors that significantly affect visual acuity (VA) and consequently, VRQoL limits this comparison. When we included patients who had discontinued treatment in this comparison, our results align with our hypothesis that patients with nAMD who had discontinued anti‐VEGF treatment experience a lower VRQoL. A similar association between treatment discontinuation and reduced quality of life has been observed in patients with chronic obstructive pulmonary disease following withdrawal of inhaled corticosteroids and subsequent decline in lung function (Calzetta et al., 2017). However, this association is not universal across all chronic diseases. For instance, studies have shown that discontinuing tyrosine kinase inhibitors in chronic myeloid leukaemia does not negatively impact quality of life (Anna Petrova et al., 2018; Schoenbeck et al., 2022). VA is known to significantly correlate with VRQoL in patients with nAMD, and prior research indicates that not all patients respond effectively to anti‐VEGF therapy, leading to a decline in vision and a corresponding reduction in VRQoL (Airody et al., 2024; Flores et al., 2021; Vu et al., 2023). Our study supports this observation, as patients who had discontinued treatment had shorter treatment durations and lower BCVA in the worse‐seeing eye. This underscores the importance of further research to optimize the efficacy of anti‐VEGF therapy and to develop alternative treatment modalities (Flores et al., 2021).

One possible reason for the low proportion of patients discontinuing treatment against medical advice in our study may be the healthcare system in Denmark. The system provides tax‐funded access to treatment and, in some cases, offers transportation to the hospital (Schmidt et al., 2019). In healthcare systems where treatment expenses are covered through patient savings, insurance, and government subsidies, such as in the Singaporean healthcare system, the situation differs (Ministry of health Singapore, 2025). A study from Singapore reported a 12‐month treatment discontinuation rate of 39.5% among nAMD patients, potentially influenced by the greater emphasis on patients' costs for treatment in combination with government subsidies (Ng et al., 2014). Conversely, treatment discontinuation of anti‐VEGF against medical advice is less frequent in the Australian and German healthcare systems, which, like the Danish system, offer some coverage for treatment and transportation, despite variations in funding, eligibility, and coverage levels (Asutralian Government Department of Health and Aged Care, 2025; Federal Ministry of Health, Germany, 2025; Holekamp et al., 2024). Geographic factors are also important to consider, as extended distances and travel times can negatively impact patient adherence, even when transportation coverage is provided (Reitan et al., 2023). An Australian study of nAMD patients reported a 10.5% permanent discontinuation rate over 6 years, with cost (2.4%) and difficulty attending appointments (0.8%) cited as reasons (Vaze et al., 2014). Other healthcare service factors, combined with patients' socioeconomic backgrounds, may also influence treatment compliance. Notably, a study in Germany revealed a 22% non‐adherence rate among nAMD patients, defining non‐adherence as a lapse in therapy of more than 100 days (Weiss et al., 2018). This demonstrates that even in healthcare systems with some coverage, non‐adherence can be high, highlighting the existence of socioeconomic and healthcare‐related factors that can affect adherence to anti‐VEGF treatment.

Poor BCVA at the baseline or lack of response to anti‐VEGF with further decline in BCVA is the most common reason for treatment discontinuation, though other factors related to adherence also warrant investigation (Borrelli et al., 2025). According to the Health Belief Model, a widely used framework for understanding health behaviours, including patient treatment adherence, an individual's health behaviours are primarily driven by their personal beliefs and perceptions regarding a disease and the perceived burdens of treatment (Grosser, 1982; Rosenstock, 1974). A previous interview‐based study with 21 Danish patients with nAMD did not identify any barriers impacting patient adherence to anti‐VEGF injections (Thinggaard et al., 2023). In contrast, our study identified several factors that contributed to patients discontinuing treatment against medical advice. Treatment with anti‐VEGF involves frequent follow‐up visits, necessitating pre‐appointment medical imaging with Optical Coherence Tomography (OCT‐imaging) and often leading to unavoidable waiting times (Thinggaard et al., 2023). These factors can make anti‐VEGF treatment a challenging preventive therapy, requiring higher effort and commitment. In a previous Danish study of older patients with nAMD, with a median age of 92.0 years (IQR 90.0–95.0 years), 13.8% of patients discontinued treatment with anti‐VEGF against medical advice (Subhi & Sørensen, 2017). The results highlight the impact of older age, which correlates with a higher prevalence of comorbidities and lower capability in maintaining treatment adherence (Okada et al., 2021). Our findings are consistent with these observations, as patients who were undergoing treatment were significantly younger and had a longer treatment duration than those who had discontinued it. Another factor that may have contributed to the high adherence was the larger proportion of patients who were undergoing treatment and did not live alone. This observation is consistent with a previous study that showed higher adherence among patients with social support for their anti‐VEGF therapy (Chang et al., 2021). The pain associated with intravitreal injections is a well‐documented burden, a finding supported by both our study and previous research (Holekamp et al., 2024; Thier et al., 2022). The risk of treatment discontinuation increases when these burdens are combined with subjective treatment dissatisfaction and low perceived benefits (Rosenstock, 1974; Thier et al., 2022).

Our study reveals that patients treated unilaterally exhibited better VRQoL, which may be explained by compensation from the healthy eye, providing a satisfactory subjective binocular vision. This point is further underscored by the fact that seven out of ten patients who had discontinued treatment against medical advice were receiving unilateral treatment. For these patients, satisfactory subjective vision may have outweighed the barriers to treatment, thereby influencing their decision to stop. On the other hand, a previous study proved that skipping treatment due to the COVID‐19 pandemic had a significant negative impact on VA (Stattin et al., 2021), which highlights the risk of severely impaired vision if treatment is discontinued against medical advice, for instance, if the fellow eye later develops nAMD.

Many approaches have been explored to minimize the burden related to intravitreal injections. A previous study suggested that a more durable treatment, leading to reduced injection frequency and visits, could lessen treatment burdens and improve patients' quality of life (Holekamp et al., 2024). A 2024 systematic review suggested that a thinner needle (30‐gauge) is associated with lower patient discomfort during intravitreal injections (Butler et al., 2024). Other studies have investigated the potential benefits of different injection techniques and the administration of pain‐minimizing medications (Raevis et al., 2019). To minimize anxiety related to intravitreal anti‐VEGF injections, some studies suggest several strategies: improved patient‐practitioner interaction (Yiallouridou et al., 2023). customizing waiting times based on patient anxiety levels (Spooner et al., 2019). and implementing feedback from patients, which is highlighted as a crucial first step in minimizing treatment‐related barriers (Yiallouridou et al., 2023). Finally, practitioners can enhance patient adherence by highlighting the negative impact of treatment discontinuation on VA (Stattin et al., 2021). This can help patients better understand the potential consequences of stopping treatment against medical advice.

## 5 STRENGTHS AND LIMITATIONS

A strength of this study is the number of patients recruited and the use of the NEI‐VFQ‐25, a widely used questionnaire that facilitates comparison with other studies. The results of this study may, however, have limited generalizability to countries with healthcare systems significantly different from the Danish system.

A limitation of this study was the omission of BCVA data close to the questionnaire response date for patients who had discontinued treatment. This limitation arises from the difficulty of collecting data from patients who had discontinued treatment. Many of these patients are elderly with limited mobility, which makes it challenging for the patients to return for additional examinations long after discharge. Similarly, information on lens status was not available in this study, as patient self‐report was deemed too unreliable, and retrieval from medical records was not feasible, particularly for patients who had discontinued treatment further in the past. Finally, the accuracy of patients' subjective vision assessments may also had been biased by unmeasured factors, such as psychological health and depression, which were common among nAMD patients (Finger et al., 2020). Recall bias regarding the dates provided may also introduce a degree of uncertainty into the study's assessment of time durations.

## 6 CONCLUSION

Patients who had discontinued treatment for nAMD report significantly lower VRQoL compared to those who were undergoing treatment. Lower BCVA in the worse‐seeing eye, older age, living alone, and unilateral treatment may be associated with treatment discontinuation. Future studies assessing VA and retinal fluid status among patients who had discontinued treatment could be valuable in advancing the understanding of this patient group. Such studies may also inform decision‐making on when it is appropriate to discontinue treatment, adopting a more holistic perspective that places patients' quality of life at the centre.

## Supporting information


**Table S1.** Crude β‐coefficients and 95% confidence intervals for the NEI‐VFQ‐25 composite score and various variables, separately for patients with nAMD who had discontinued treatment with anti‐VEGF (*n* = 172; 116 unilateral, 54 bilateral) and patients who were undergoing treatment (*n* = 365; 210 unilateral, 146 bilateral).
