# Supplementary material for: Characterizing and assessing vision‐related quality of life among patients discontinued treatment for neovascular age‐related macular degeneration
Source: Acta Ophthalmol. 2025 Dec 15;104(4):e406–15. doi: 10.1111/aos.70044 (PMC13166389; doi:10.1111/aos.70044)
Supplement: Supplementary file 1 — Table S1. Crude β‐coefficients and 95% confidence intervals for the NEI‐VFQ‐25 composite score and various variables, separately for patients with nAMD who had discontinued treatment with anti‐VEGF (n = 172; 116 unilateral, 54 bilateral) and patients who were undergoing treatment (n = 365; 210 unilateral, 146 bilateral). [file AOS-104-e406-s001.docx]

**Supplementary table 1:** Crude β-coefficients and 95% confidence intervals for the NEI-VFQ-25 composite score and various variables, separately for patients with nAMD who had discontinued treatment with anti-VEGF (n=172; 116 unilateral, 54 bilateral) and patients who were undergoing treatment (n=365; 210 unilateral, 146 bilateral).

|  | Crude β-coefficient [95% CI] | |
| --- | --- | --- |
| Variable | Discontinued treatment | Undergoing treatment |
| Age | -1.10 [-1.59, -0.60] | -0.43 [-0.72, -0.13] |
| Biological sex |  |  |
| Female | Ref | Ref |
| Male | 10.01 [2.69, 17.33] | 5.62 [1.71, 9.54] |
| BCVA |  |  |
| Best-seeing eye | 44.48 [36.10, 52.86] | 36.34 [29.43, 43.24] |
| Worse-seeing eye | 32.21 [23.32, 41.10] | 26.32 [20.22, 32.43] |
| Marital status |  |  |
| Married | Ref | Ref |
| Widow/widower | -16.53 [-24.89, -8.17] | -5.72 [-10.24, -1.20] |
| Divorced/never married/other | -0.77 [-10.07, 8.52] | 2.52 [-2.93, 7.97] |
| Living situation |  |  |
| Not living alone | Ref | Ref |
| Living alone | -10.21 [-17.63, -2.79] | -2.99 [-7.11, 1.15] |
| Treatment laterality |  |  |
| Unilateral | Ref | Ref |
| Bilateral | -11.39 [-19.35, -3.44] | -5.95 [-9.93, -1.98] |
| Home-to-treatment travel time |  |  |
| <30 min | Ref | Ref |
| ≥30 min | -3.36 [-10.96, 4.25] | -1.68 [-5.83, 2.47] |
| Treatment duration | - | -0.78 [-1.35, -0.20] |
| Duration since the last injection | -1.65 [-3.05, -0.25] | - |

Footnotes: NEI-VFQ-25 = national eye institute visual function questionnaire-25; nAMD = neovascular age-related macular degeneration; Anti-VEGF = anti-vascular endothelial growth factor; CI = confidence interval; Ref = reference; BCVA = best corrected visual acuity (Snellen).
